# BARRIERS, FACILITATORS, BENEFITS, AND ENJOYMENT OF PHYSICAL ACTIVITY IN MIDDLE-AGED HISPANIC MEN

**DOI:** 10.1093/geroni/igz038.730

**Published:** 2019-11-08

**Authors:** Ferdinand Delgado, Cheryl DerAnanian, Sonia Vega-Lopez, Shandel Vega-Soto, Hector Valdez, Steven Hooker

**Affiliations:** 1 Arizona State University, Phoenix, Arizona, United States; 2 Arizona State University, Phoenix, United States; 3 San Diego State University, San Diego, California, United States

## Abstract

Little is known about middle-aged Hispanic men’s perceptions of physical activity (PA). Purpose: To examine perceived barriers, facilitators, and benefits of PA and what types of PA are enjoyable. Methods: Seven focus groups (FGs) were conducted with middle-aged Hispanic men (mean age 51.6±6.1 years; n=32) who primarily self-identified as Mexican (78.1%). All FGs were audio-recorded, transcribed, and translated into English. A grounded theory approach was used to identify themes. Results: Competing responsibilities (N=7 FGs), laziness/apathy (N=6 FGs), and a lack of habit/routine (N=5 FGs) were identified as the primary barriers to PA. Jobs were perceived as a PA barrier because of physical labor involved, long hours, having multiple jobs, and/or an inconsistent schedule. Laziness/apathy involved the notion that people have time to exercise, but choose not to. Not having a routine resulted in procrastination and not achieving exercise goals. Social support was the primary facilitator to PA, which included having a friend (N=5 FGs), family member (N=5 FGs), or spouse/partner (N=6 FGs) with whom they could participate in PA. Sports were mentioned in all FGs as enjoyable, including soccer (N=5 FGs), basketball (N=5 FGs), tennis/racquetball/handball (N=3 FGs), and baseball (N=2 FGs). Walking/running (N=6 FGs) and hiking (N=5 FGs) were also considered enjoyable. The primary perceived benefits of PA included increased energy after exercising (N=6 FGs) and improved overall health (N=6 FGs). Conclusion: Hispanic men realize the importance of PA and what may be hindrances or facilitators to increasing PA. Funded by the National Institute on Aging (R21 AG050084-01A1).

